# Creation of equal-spin triplet superconductivity at the Al/EuS interface

**DOI:** 10.1038/s41467-018-07597-w

**Published:** 2018-12-07

**Authors:** S. Diesch, P. Machon, M. Wolz, C. Sürgers, D. Beckmann, W. Belzig, E. Scheer

**Affiliations:** 1 Department of Physics, University of Konstanz, Universitätsstraße 10, D-78457 Konstanz, Germany; 2 Physikalisches Institut, Karlsruhe Institute of Technology (KIT), Wolfgang Gaede Straße 1, D-76131 Karlsruhe, Germany; 3 Institute of Nanotechnology, Karlsruhe Institute of Technology (KIT), Hermann-von-Helmholtz-Platz 1, D-76344 Eggenstein-Leopoldshafen, Germany

## Abstract

In conventional superconductors, electrons of opposite spins are bound into Cooper pairs. However, when the superconductor is in contact with a non-uniformly ordered ferromagnet, an exotic type of superconductivity can appear at the interface, with electrons bound into three possible spin-triplet states. Triplet pairs with equal spin play a vital role in low-dissipation spintronics. Despite the observation of supercurrents through ferromagnets, spectroscopic evidence for the existence of equal-spin triplet pairs is still missing. Here we show a theoretical model that reveals a characteristic gap structure in the quasiparticle density of states which provides a unique signature for the presence of equal-spin triplet pairs. By scanning tunnelling spectroscopy we measure the local density of states to reveal the spin configuration of triplet pairs. We demonstrate that the Al/EuS interface causes strong and tunable spin-mixing by virtue of its spin-dependent transmission.

## Abstract (web summary)

Spectroscopic evidence of equal-spin triplet Cooper pairs is still missing so far. Here, Diesch et al. propose a unique signature for the presence of equal-spin triplet pairs and experimentally reveal the spin configuration of triplet pairs at the Al/EuS interface.

## Introduction

Hybrid superconductor-ferromagnet (S/F) heterostructures are fundamental building blocks for next-generation, ultralow power computers. In these devices, Cooper pairs of equal spin would carry spin information without dissipation^1,2^, reducing power consumption by several orders of magnitude^3^. Due to these promising applications, such exotic electron states have attracted considerable interest in recent years: after the initial prediction of triplet superconductivity in conventional s-wave superconductors, the theory has been elaborated to cover several scenarios, including multilayers and microscale devices^4–10^. The Pauli principle can be fulfilled for both singlet and triplet Cooper pairs by adjusting the symmetry in the time argument. Thus, spin triplet pairs in s-wave superconductors with even spatial symmetry must have a pair correlation function that is odd in the time argument, called odd frequency spin triplets^5,11^. Triplet pairs are created at interfaces between superconductors and ferromagnetic materials, and their properties have been studied theoretically intensively^12,13^. When a single magnetisation direction is present, solely *m* = 0 triplet pairs $\left( {{\textstyle{1 \over {\sqrt 2 }}}\left[ {\left| { \uparrow \downarrow } \right\rangle + \left| { \downarrow \uparrow } \right\rangle } \right]} \right)$ are created by spin-mixing, i.e., spin-dependent phase shifts for the electrons scattering at this interface^11^. These triplet pairs can be converted into Cooper pairs with parallel spins, ${m = \pm 1\left( \left| { \uparrow \uparrow } \right\rangle {\mathrm{/}}\left| { \downarrow \downarrow } \right\rangle \right)}$ by a second, non-collinear magnetisation direction defining a new spin quantisation axis^10^. We will refer to these different kinds of pairings as mixed-spin (*m* = 0) or equal-spin (*m* = ±1) state.

Experimentally, the existence of equal-spin pairing has been shown by measuring the penetration depth of the supercurrent in ferromagnetic materials^14–16^, where the term long-ranged spin-triplet superconductivity has been coined. More experimental indication for spin-triplet superconductivity has been reported from measurements of the critical temperature on S/F/F’ spin valves^17–20^, from measurements of the supercurrent through S/F/F′/F″/S junctions as a function of the relative magnetisation directions of the three ferromagnetic layers^21,22^ and by studies of the paramagnetic Meissner effect in S/F bilayers^23^. These works, however, do not consider non-collinear magnetisations and can therefore, in principle, not distinguish different types of spin-triplet superconductivity. Obtaining experimental support for the creation of triplet pairs from measurements of the quasiparticle density of states by scanning tunnelling spectroscopy (STS) has been suggested^24,25^. In a previous study, STS has been performed on Nb superconducting films proximity coupled to the chiral ferromagnet Ho^26^. In that work a Nb/Ho bilayer has been probed from the superconducting side and the obtained spectra were compared to a specialised theoretical model relying on the chiral magnetic state of Ho and pinning effects occurring at the Nb/Ho interface. Both zero-bias peaks and double peaks in the spectra were observed and interpreted as signatures of triplet superconductivity consistent with theory^25^. However, only very qualitative agreement between experimental and theoretical spectra could be achieved, most likely because the full boundary conditions were not used^27^.

As a result, it has not been revealed whether these triplets are equal-spin or mixed-spin pairs due to a missing unique signature, which would allow to distinguish between these two possibilities experimentally. Furthermore, the influence of the spin-dependent phase shifts are expected to be much weaker on the S side of the interface, resulting in only small amplitudes of the subgap features. To address these two issues, we study here the local density of states (LDOS) of an S/FI/N trilayer, where FI is a ferromagnetic insulator with non-collinear magnetisation. We first predict theoretically the formation of a small spectral gap, henceforth called triplet gap, in the LDOS and show how it is related to the creation of equal-spin triplet pairs. We then support the theory through experimental STS studies on the normal side of the trilayer. To our knowledge, such a method for clearly identifying the triplet states spectroscopically has not been reported before, neither theoretically nor experimentally. The fact that equal-spin and mixed-spin states result in distinctly different structures in the LDOS is a novel observation. The triplet gap develops around zero energy, resulting in a symmetric double-peak structure around zero-bias voltage in the LDOS. The width of the triplet gap monotonically depends on the ratio of equal-spin to mixed-spin states in the pairing amplitude. Such a formation of an additional gap within the superconducting gap, solely depending on the magnetic structure in the proximity of the superconductor represents a new signature of equal-spin triplet Cooper pairs. Our experimental evidence presented below strongly supports the formation of an equal-spin triplet state, thus making a strong case to pursue superconducting spintronics.

## Results

### Circuit theory

**Fig. 1:**
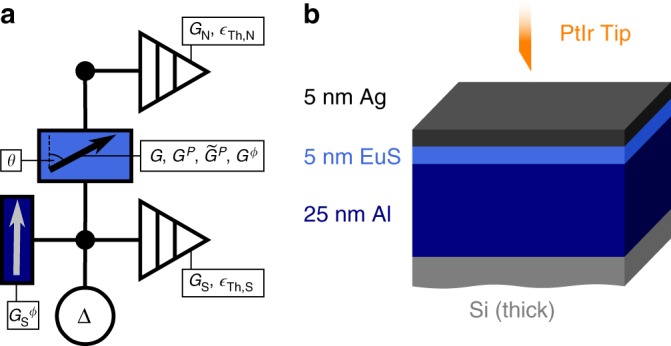
Illustration of the theoretical and experimental setup. **a** Circuit diagram representing the theoretical model. A superconducting S-node with a Δ-pseudo-terminal is connected to a normal conducting N-node (both with conductances *G*_S/N_ and Thouless energies $\epsilon _{{\mathrm{N/S}}}$) via a tunnelling connector. The connector has the spin-dependent parameters $\tilde G^P,G^P$ and *G*^*ϕ*^, which depend on the relative magnetisation direction *θ* to the spin-dependent *G*^*ϕ*^ term of the S-node. The relative angle *θ* between those magnetisations is the main free parameter in our fits. **b** Schematic of the tunnel contact. A PtIr tip (normal metal) is brought into tunnel contact with a trilayer sample of an EuS layer sandwiched between a normal conducting Ag and a superconducting Al film

In our theory based on the language of circuit theory^27–33^, a ferromagnetic insulator separating an s-wave superconductor and a normal metal can be represented by the circuit diagram depicted in Fig. 1a. Each conducting layer is represented by one node, characterised by its conductance *G*_N/S_ (the index N/S labels the normal/superconductor side) and its size-dependent Thouless energy $\epsilon _{{\mathrm{Th,N/S}}}$. The superconducting layer is specified by the pseudo terminal characterised by the pair potential Δ, which constitutes a source of coherence that has to meet the self-consistency relation^34^. The ferromagnetic insulator is described by a connector representing a tunnel barrier with the conductance *G*_T_. The ferromagnetic nature is accounted for by parameters *G*^*P*^ and $\tilde G^P$ resulting from the spin polarisation of the tunnel probabilities^27,32^, and the so-called spin-mixing term *G*^*ϕ*^^30^, which all depend on the respective magnetisation directions. We use two *G*^*ϕ*^ terms, one in the ferromagnetic connector and one in the superconducting node, to account for the magnetic texture in the system (which we will discuss later). One advantage of the circuit theory approach is that it starts from very basic concepts and can easily be extended to describe many other realisations of S, F, N heterostructures. Note that in order to realistically represent the investigated circuit with the specific model chosen here, the layer thicknesses must not exceed the coherence length of the superconductor.

**Fig. 2:**
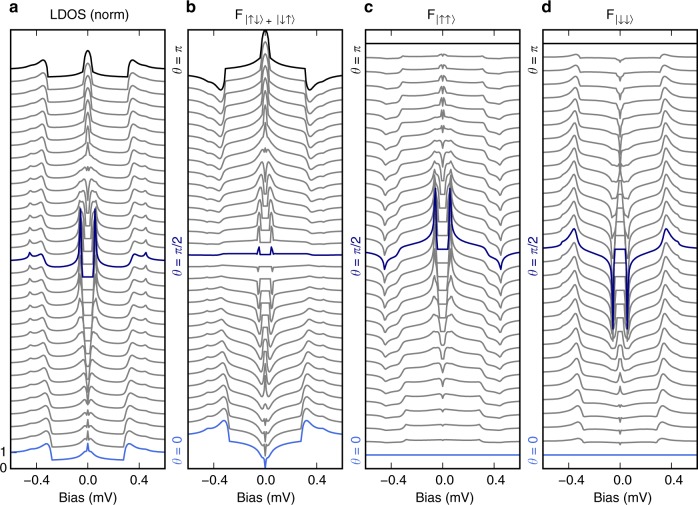
Dependence of the superconducting properties on the magnetic configuration. **a** Evolution of the local density of states (LDOS) as a function of the relative angle *θ* between the magnetisation of the ferromagnetic connector and the *G*^*ϕ*^ term of the S-node, showing zero-bias peaks with varying amplitude for parallel (*θ* = 0) and anti-parallel (*θ* = *π*) alignment and the appearance of the triplet gap around perpendicular alignment. **b** Pair amplitude of the mixed-spin and **c**, **d** the equal-spin components as a function of *θ*. The mixed-spin component is prominent for *θ* ≈ 0 and *θ* ≈ *π* and almost vanishes around *θ* = *π*/2, whereas for the equal-spin component it is opposite. All curves have been offset vertically for better visibility

The circuit diagram in Fig. 1a represents the discretised version of the Usadel equation^28,35^ and has to be expanded with a spin-dependent boundary condition, details of which are shown in the Methods section and a full derivation of which can be found in previous publications^27,32,33^. Solving the Usadel equation (see Eq. (1) in the Methods section) allows us to calculate the LDOS in the N-node. Changing the direction of magnetisation in the ferromagnetic connector parametrised by the angle *θ* between the magnetisations of the ferromagnetic insulator’s interior and the interface spins, results in an evolution of features inside the gap shown in Fig. 2a. For *θ* close to 0 and *π*, the LDOS shows a peak at zero bias (corresponding to the Fermi energy). For all other angles, i.e., situations where not all magnetisation directions are collinear, a gap opens symmetrically centered at the Fermi energy. This non-collinear orientations of magnetisations in the system has been identified as a mandatory prerequisite of equal-spin triplet pairing^11^. Accordingly, the gap is most pronounced at *θ* = *π*/2, corresponding to a maximal equal-spin pairing. This is visualised in Fig. 2c, d, where the equal-spin triplet components of the anomalous Green’s functions of the superconductor are plotted in the *z*-basis projected onto the magnetisation direction of the S-node **m**_S_. It is important to stress that the equal-spin triplet pairing $F_{\left| { \uparrow \uparrow } \right\rangle /\left| { \downarrow \downarrow } \right\rangle }$ has a distinctly different energy dependence than the mixed-spin pairing $F_{\left| { \uparrow \downarrow } \right\rangle + \left| { \downarrow \uparrow } \right\rangle }$ shown in Fig. 2b. Hence, a full interpretation of the LDOS requires a simultaneous consideration of the energy-dependent pair amplitudes.

### Scanning tunnelling spectroscopy

From the calculated LDOS, the theoretical differential conductance d*I*/d*V* can be calculated by including experimental parameters like non-zero temperature and amplitude of the voltage modulation added to the bias in order to perform lock-in measurements. These calculated curves are compared to d*I*/d*V* tunnel spectra, measured in lock-in technique between a normal metal tip and an Al/EuS/Ag trilayer sample (see Fig. 1b and Methods section) in a scanning tunnelling microscope (STM) at 290 mK, far below the superconducting critical temperature of the Al layer (*T*_c_ = 1.7 K). As we discuss in Supplementary Note 2, there is strong evidence for the formation of an oxide layer at the interface between Al and EuS. As we will argue below, this oxide layer might be important for the formation of the non-collinear magnetisation arrangement, which itself is crucial for the formation of the triplet pairs. The changing direction of magnetisation in the tunnel connector is experimentally realised by exposing the sample to an external magnetic field parallel to the sample plane.

### Spatial dependence of subgap features

**Fig. 3:**
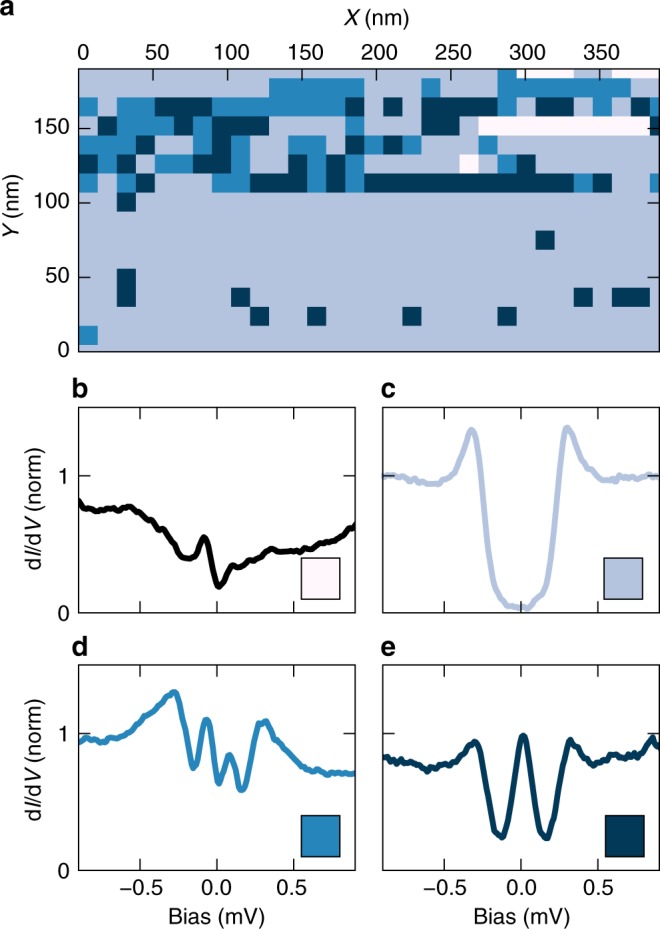
Spatial dependence of the differential conductance. **a** Map of locations on the sample surface where different types of spectra shown in **b**–**e** have been observed. The different colours in **a** correspond to the different types of spectra observed and match the spectra shown in **b**–**e**. The shape of the spectra changes on length scales corresponding to the grain size of the EuS films (8–18 nm)^37^. Data were recorded on sample EuS-3

In order to characterise the trilayer film sample, we record tunnel spectra (see Supplementary Note 1) by scanning the tip over the sample with a step size of 12.5 nm. In Fig. 3 we show a colour-coded map of typical d*I*/d*V* spectra. All spectra are normalised to the conductance value *G*_b_ far outside the gap. We categorised the spectra in four distinct groups, each characterised by a specific shape (Fig. 3b–e). Category (b) corresponds to tip locations where the tunnel contact is too noisy for spectroscopy or where superconductivity is being suppressed. Spectra of these types are only rarely observed. We attribute these spectra to surface contamination or defects in the film. Category (c) shows a spectrum with a hard gap as known from BCS theory, which is the result known for spin-independent tunnelling and is seen in all Al/Ag bilayer reference samples (see Supplementary Fig. 1). The gap size Δ ≈ 250 ± 20 μeV is slightly enhanced with respect to the bulk value of Δ_0_ = 180 μeV as usual for thin Al films^36^. The most relevant spectra for this work are of type (d) and (e), in the following referred to as triplet gap and zero-bias peak spectra, respectively. They show coherence peaks at the gap edge similar to BCS theory, however, inside the gap the d*I*/d*V* remains finite everywhere with a minimum value ≈0.25–0.5*G*_b_. The triplet gap spectra (d) feature two peaks located symmetrically around zero bias, which are not necessarily symmetric in height. Category (e) shows a maximum at zero bias the amplitude of which can be larger than *G*_b_. While the majority of the spectra recorded on all measured samples show the BCS-like spectra (c), some areas show reproducible clusters of zero-bias peaks or triplet-gap features when addressing the same spot repeatedly. The fact that spectra can swap from triplet gap to zero-bias peak and to BCS like within one pixel reveals that the phenomenon responsible for the nature of the features inside the gap can change on short length scales, corresponding to, e.g., the grain size of the EuS thin film (8–18 nm)^37^.

### Non-collinear magnetic model

**Fig. 4:**
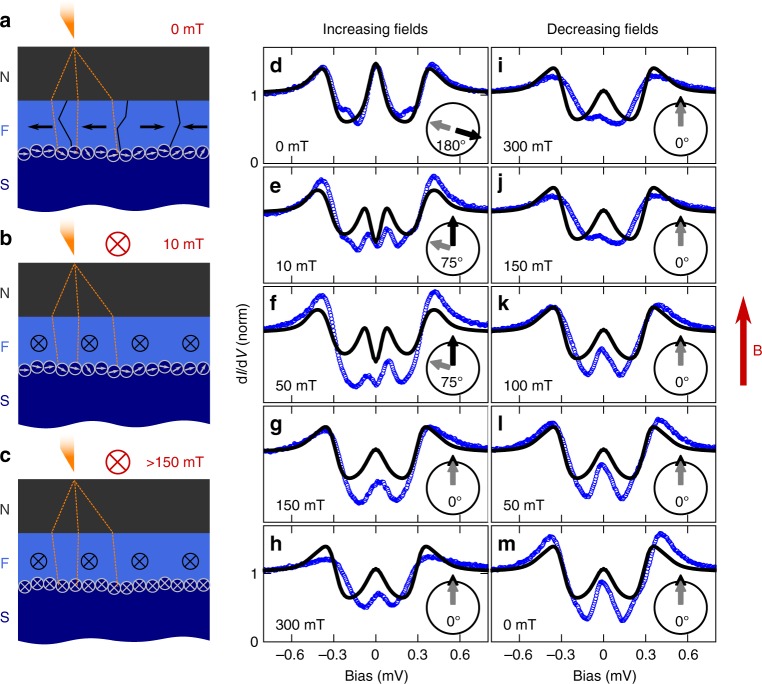
Model of the magnetisation behaviour of the EuS layer. **a** The sample in the zero-field cooled (ZFC) state consists of magnetically soft domains (black arrows) with an overall magnetic moment that is random in direction, and harder interface magnetic moments (grey arrows). **b** The internal domains are expected to follow the external magnetic field (directed into the plane of projection) more readily, aligning at smaller magnetic fields. **c** The interface moments follow the applied field only for higher field values. **d**–**m** Experimental d*I*/d*V* spectra (blue circles) recorded for the same tunnel contact in varying magnetic fields at 290 mK and theoretical spectra (black curves) fitted to the data. A full up and down sweep is performed to show that the observed curves depend on the magnetisation behaviour of the F layer. The black and grey arrows indicate the fitted relative angle between the different magnetisations. Direction of the external field is indicated in red. Data were recorded on sample EuS-1

To explain our findings we propose the following model: According to our theoretical studies, the appearance of spectra with triplet gap features corresponds to areas with at least two magnetisation directions which are non-collinear. We assume these two distinct magnetic areas are given by the bulk EuS film on the one hand, and by the interface to the Al layer on the other hand (Fig. 4a–c), as we will explain in more detail below and in Supplementary Note 3. Second, the ferromagnetic interface must provide some degree of spin mixing between similarly oriented magnetic domains. These are two non-trivial requirements explaining why only a fraction of all spectra reveals these features.

The dominance of BCS-like spectra indicates that the domain size of the ferromagnetic film is small. As wide areas of the EuS film have proven to be nanocrystalline in transmission electron microscopy (TEM) measurements (see Supplementary Fig. 3), the large number of domains probed simultaneously results in a minimised average magnetisation. Areas that do show zero-bias peak and triplet-gap features thus hint at locally enlarged domain sizes, thereby reducing the effective number of domains being studied at one spot and resulting in a net magnetic moment. Such an extreme sensitivity of the superconducting state to the local domain structure is well known^12^. As no voids or areas with reduced thickness of the EuS film are observed in the TEM images, we consider the possibility, that no ferromagnetic material was present in large parts of the trilayer, to be unlikely.

### Magnetic field dependence

To further verify this model, we performed STS measurements with applied magnetic field, see Fig. 4. We assume that the magnetic configuration of a sample in the zero-field cooled (ZFC) state consists of domains with independent magnetisations pointing in random directions^38^ (black arrows) and of moments found at the interface of the ferromagnet, that do not necessarily align with the direction of magnetisation of the underlying bulk domains^26^ (grey arrows). Once exposed to external magnetic fields, those interface moments follow the direction of the external field only at higher fields compared to the magnetically softer bulk moments. These interface moments can be magnetically harder due to a local variation of the interface^39,40^ or surface effects^41^. The exact nature of the interface moments is irrelevant for the functional principle of the creation of spin-triplet pairs by non-collinear magnetisation. As mentioned above, we observed an oxide layer at the interface between Al and EuS. In Supplementary Note 2, we show that this layer contains grains of EuO. EuO is a ferromagnet with a higher Curie temperature than EuS. Together with the small size of the grains they may be magnetically harder than the bulk EuS film and may help creating locally a non-collinear magnetisation configuration.

We can use the different coercive fields of the oxide interface and the bulk moments of the EuS layer to control the angle between them by applying an external magnetic field (Fig. 4a–c). In order to better understand the relationship between the features inside the gap and the magnetic properties of the F/S system, we show a complete up and down sweep of the in-plane magnetic field at a position on the sample, where a zero-bias peak appears in the ZFC state (for the evolution of a triplet gap feature in magnetic fields see Supplementary Note 4 and Supplementary Fig. 9), and we fit differential conductance spectra calculated using the model described above to the experimental data (Fig. 4d–m). This fitting is done under the constraint that only the relative magnetisation angle *θ* between the oxide interface moments and the bulk moments can change between different set points of the external field. The parameters characteristic for the sample geometry $\left( {G_{{\mathrm{S/N}}},G_{{\mathrm{S/N}}}^\phi ,P} \right)$ and the materials used (*T*_c_ = 1.84 K, and derived from these self-consistently Δ = 280 μeV), were fit to the zero-field spectrum and then kept constant. We note, that the quality of the fits could be substantially improved by varying these parameters individually for every field independently, which would, however, not be justified by physical arguments. Due to the complexity of the model there are several combinations of spin-dependent parameters (see Supplementary Note 6 and Supplementary Table 2), which fit the experimental spectra almost equally well. However, all these parameter sets correspond to the same evolution of the relevant physical properties (see Supplementary Figs. 11 and 12), i.e., the same magnetisation configurations. The solutions share a strong-induced exchange field in the Al (here measured by $G_{\mathrm{S}}^\phi$), and a large spin-polarisation *P*_*n*_ of the tunnel current of at least 60%. For distinctly smaller values of either $G_{\mathrm{S}}^\phi$ or *P*_*n*_ the hallmarks of equal-spin triplets vanish. The material choice is thus crucial for the creation of equal-spin triplet pairs.

The observed field dependence can be consistently interpreted when assuming the tip to be located at an area on the sample, where the magnetic configuration in the ZFC state is anti-parallel. Anti-parallel configurations between interface moments and bulk magnetisation might be energetically favoured because of their reduced stray-field. Microscopically this could be realised by a magnetically harder layer of interface moments^41^ or by the formation of a ferromagnetic oxidised state of EuS at the EuS/Al interface (see Supplementary Note 2). In the circuit theory model, this anti-parallel configuration results in a strong zero-bias peak in the LDOS, which corresponds to the creation of mixed-spin triplet pairs as visible by the peak in the mixed-spin pairing amplitude (Fig. 2b). The experimental data follow this prediction closely. As the magnetic field is increased, the bulk magnetisation readily follows the field direction, and at 10 mT already the angle between the interface moments (grey arrows) and the bulk magnetisation (black arrows) is decreased. Fitting this misorientation to our experimental data results in an angle of 75°, which means that the initial bulk magnetisation was rotated in-plane by 105° (Fig. 4e). In our theory model, this rotation from anti-parallel to non-collinear magnetisations opens up a gap in the LDOS, which directly corresponds to the creation of equal-spin triplet Cooper pairs, as signalled by the increasing equal-spin triplet pairing amplitudes (Fig. 2c, d). The experimental data clearly reflect this trend. As the magnetic field is further increased, the oxide interface moments finally also follow the field direction at around 150 mT. The triplet gap and its confining double peaks disappear to reveal again a zero-bias peak, corresponding to collinear magnetisations according to our fits. This behaviour, the appearance, disappearance, and reappearance of a double peak around zero bias, cannot be explained by a simple Zeeman shift of the LDOS (see Supplementary Note 8 and Supplementary Fig. 11). Further increasing the external field does not substantially change the features inside the gap, but suppression of the superconducting gap by the magnetic field starts at around |*B*| ≈ 300 mT. As expected, here the theoretical model does not describe the experimental spectra any more, as this suppression is most likely due to the onset of orbital depairing, which would requires yet another fit parameter in the theory. As we decrease the external field, no triplet gap opens up and no double peaks reappear, supporting our assumption that we started with a magnetic configuration in the anti-parallel state, which we cannot recover by decreasing the field. However, the zero-bias peak starts reappearing at around 300 mT and is fully developed at 100 mT. This model for the as-cooled magnetisation configuration also explains why finding such a transition is so rare—most in-gap features show a much less pronounced field dependence under varying the external magnetic field.

In conclusion, we have shown combined theoretical and experimental evidence that a non-collinear magnetic configuration of the S/FI interface leads to the appearance of a novel type of gap in the superconducting density of states. This triplet gap is closely related to the creation of equal-spin triplet Cooper pairs because it goes along with a significant increase of the equal-spin triplet pair amplitudes. Zero-bias peaks, contrary to earlier claims, do not hallmark equal-spin triplets, but short-ranged mixed-spin triplets. By selectively tuning the relative magnetisation direction between magnetic moments trapped at the interface and the softer magnetisation of the bulk domains, we are able to significantly influence the LDOS of the system. Our experiments provide spectroscopic evidence for the superconducting state induced by an FI interlayer with non-collinear magnetic texture. Here, the FI is realized by EuS that not only provides high spin polarisation and effective creation of spin splitting in the superconductor Al, but also builds up an oxide layer between the FI and the superconductor.

Our study also reveals that local variation from a collinear magnetisation arrangement is mandatory to form equal-spin triplet Cooper pairs. The FI thus fulfills several functions: By coupling it to the superconductor it creates spin-triplet correlations, promotes spin-dependent tunnelling of the pair amplitude, as well as non-collinear texture on small length scales. In EuS the texture is given by the grain size and by the formation of an oxide layer between EuS and Al. The texture can be elegantly tuned by an external magnetic field, and thereby the magnitude of the spin-polarised Cooper pairs can be adjusted, thus opening up the possibility for controlling dissipation-less transport of spin information in spintronics devices.

## Methods

### Circuit theory

The spin-dependent boundary condition of the Usadel equation for an arbitrary contact depends on the transmission probability *T*_*n*_, polarisation *P*_*n*_, the spin mixing angle *ϕ*_*n*_^42^ and the magnetisation axes with unit vector **m**_*n*_, wherein the index *n* labels the transport channels. The number of channels depends on the size and the shape of the tunnel contact. For simplicity, we assume a smooth contact plane, thus the number of channels *N* is given as $N = Ak_{\mathrm{F}}^2{\mathrm{/}}(4\pi )$, where *A* is the tunnelling contact area and *k*_F_ the Fermi wave vector.

We work in the tunnel limit $\left( {T_n \ll 1} \right)$ and assume small spin mixing $\left( {\phi _n \ll 1} \right)$. In this case, the combination of the discrete Usadel equation and the boundary condition leads to two coupled equations, one for each node^29^, expressing the matrix current conservation

1 $$\check I_{\mathrm S \rightarrow \mathrm N} + \check I_{\mathrm{N}}^L = 0 = \check I_{\mathrm N \rightarrow \mathrm S} + \check I_{\mathrm {S}}^L\,.$$

The matrix currents are given by

$$\check {I}_{\mathrm{N}}^L 	 = \left[-i \epsilon \frac{G_{\mathrm{N}}}{\epsilon_{\mathrm{Th,N}}}\tau_3,\check{G}_{\mathrm{N}}\right],\quad\check{I}_{\mathrm{S}}^L = \left[x` \frac{G_{\mathrm{S}}}{\epsilon_{\mathrm{Th,S}}}\left(-i\epsilon\tau_3+\check{\Delta}\right) -i G^{\phi}_{\mathrm{S}} \check{\kappa}_{\mathrm{S}} ,\check{G}_{\mathrm{S}}\right]\\ \check{I}_{{\mathrm{S}} \rightarrow {\mathrm{N}}} 	= \frac{1}{2} \left[\frac{G_{\mathrm{T}}}{2} \left\{\check{G}_{\mathrm{S}}, \check{\kappa}\right\} \check{\kappa} + \frac{\tilde{G}^P}{2}\left[\check{G}_{\mathrm{S}}, \check{\kappa}\right]\check{\kappa} + G^{P}\left\{\check{G}_{\mathrm{S}}, \check{\kappa}\right\} -iG^{\phi}\check{\kappa}, \check{G}_{\mathrm{N}}\right] \\ \check{I}_{{\mathrm{N}} \rightarrow {\mathrm{S}}} 	 = \frac 12 \left [\frac{G_{\mathrm{T}}}{2} \left\{\check{G}_{\mathrm{N}}, \check{\kappa} \right\}\check{\kappa} + \frac{\tilde{G}^P}{2}\left[\check{G}_{\mathrm{N}}, \check{\kappa}\right]\check{\kappa} + G^P \left\{\check{G}_{\mathrm{N}}, \check{\kappa}\right\} - i G^{\phi}\check{\kappa}, \check{G}_{\mathrm{S}}\right].$$

The equations are closed by demanding the normalisation conditions $\check{G}_{\mathrm{S}}^2=1=\check{G}_{\mathrm{N}}^2$. The conductances are defined from the experimental interface parameters. The spin-independent parameter is $G_{\mathrm{T}} = 2\mathop {\sum}\nolimits_n {\kern 1pt} T_n$ and the spin-dependent parameters are $\tilde G^P = 2\mathop {\sum}\nolimits_n {\kern 1pt} T_n\sqrt {1 - P_n^2}$, $G^P = \mathop {\sum}\nolimits_n {\kern 1pt} T_nP_n$, and $G^\phi = 2\mathop {\sum}\nolimits_n {\kern 1pt} \delta \phi _n$. We defined $\check{\kappa} = \tau _3 \otimes {\bf{m}}\sigma$, with *τ*_*i*_ and *σ*_*i*_ being Pauli matrices in Nambu and spin space, respectively. For the theoretical curves shown in Figs. 2 and 4, the following parameters were used: $G_{\mathrm{S}}{\mathrm{/}}\left( {G_{\mathrm{T}}\epsilon _{{\mathrm{Th,S}}}} \right)$ = 4.1/(*k*_B_*T*_c_), $G_{\mathrm{S}}^\phi {\mathrm{/}}G_{\mathrm{T}} = 5$, $G_{\mathrm{N}}{\mathrm{/}}\left( {G_{\mathrm{T}}\epsilon _{{\mathrm{Th,S}}}} \right)$ = 0.07/(*k*_B_*T*_c_), *G*^*ϕ*^/*G*_T_ = −0.061, and *P*_*n*_ = 0.6.

The directions of the respective magnetisation directions are denoted by **m**. The gap matrix is defined by $\check{\rm{\Delta}} = {\mathrm{\Delta }}\tau _1$. Note that the LDOS measured in the experiment does not depend on the lateral system size and, hence, Eq. (1) can be normalised e.g. by the conductance of the connector *G*_T_, thus giving a solution independent of the size of the interface area. The leakage parameters can be further related to sample parameters via $G_{{\mathrm{N/S}}}{\mathrm{/}}\epsilon _{{\mathrm{Th,N/S}}}NG_0$ = $8\pi ^2N_{0,{\mathrm{S/N}}}d_{{\mathrm{S/N}}}{\mathrm{/}}k_{{\mathrm{F,S/N}}}^2$ ~ $d_{{\mathrm{S,N}}}{\mathrm{/}}\hbar v_{{\mathrm{F,S/N}}}$ with the thickness of the layer *d*, the density of states at the Fermi energy *N*_0,S/N_ and the Fermi wave number/velocity *k*_F,S/N_, *v*_F,S/N_. We also note, that the order parameter Δ has been calculated self-consistently according to the standard BCS relations.

### Sample fabrication and characterisation

For samples EuS-1 to EuS-4, a 25 nm Al layer, followed by an EuS film of varying thickness, and a 5 nm capping Ag layer are deposited by e-beam evaporation on a silicon (111) chip. Sample EuS-5 had a thinner Al layer of ≈10 nm. For this work, five batches of samples were fabricated, with the exact parameters shown in Supplementary Table 1. The substrate and the sample holder are cooled to below 100 K using liquid nitrogen to grow an Al film of homogeneous thickness with relatively small grain size. At 25 nm, the surface RMS roughness of Al is <0.6 nm, thus forming a smooth surface for the subsequent EuS and Ag layers. The EuS layer is evaporated onto the substrate at higher temperatures to provide a compound with a Curie temperature (see Supplementary Fig. 5) close to the value reported in the literature (*T*_Curie_ = 16.7 K^43^). At room temperature, EuS is semiconducting with an indirect energy gap, the conduction band minimum at 300 K is 1.64 eV^44^. At cryogenic temperatures, EuS is insulating with resistivity of around *ρ* = 10^4^ Ω cm for high-quality single crystals^45^. Disorder at the atomic level and unintentional doping can lower this resistivity by several orders of magnitude and at the same time increase the Curie temperature (as observed by SQUID magnetometry measurements in Supplementary Fig. 6) due to interactions between charge carriers and the Eu^2+^ ions^45^. Because of the presence of an additional insulating oxide film under the EuS layer (see Supplementary Figs. 3 and 4), we assume the ferromagnetic insulator layer to have very low conductance, i.e. enabling only tunnel transport. The surface RMS roughness of this oxide layer is ≈0.52 nm for line profiles recorded on TEM lamellas.

The deposition rate for the EuS film is 0.01 nm s^−1^, and the final thickness of the film is varied from sample to sample (each chip holding samples with four different thicknesses) using a sample holder with a movable shutter that allows parts of the chip to be covered during evaporation. The surface RMS roughness of the EuS layer is ≈0.59 nm for line profiles recorded on TEM lamellas. However, during STS measurements we often observe variations of the spectra taken on different locations on the same sample to be more pronounced than the differences between the various film thicknesses. This supports our theory that the EuS film has only vanishing magnetic influence on a proximitised superconductor when nanocrystalline, whereas regions with more uniformly magnetised domain clusters or locally enlarged domains can strongly influence the superconducting state. Thus, the lateral extent of the magnetically ordered region seems to play a much larger role than the film thickness.

Some of our EuS films were found to be conductive, allowing for a proximity coupling between Al and Ag that is much stronger than anticipated. These samples generally displayed triplet gap features only for very few locations on the film, or often not at all. Al is a suitable superconductor for this study because of its long spin relaxation time^46^. Thus, electrons that have experienced spin-mixing at the EuS interface can carry that information far into the superconductor. The bulk critical field of Al (*μ*_0_*H*_c_ = 10 mT) is significantly increased in thin films (see Supplementary Fig. 2). For in-plane fields at 25 nm thickness it is around 800 mT^46^. In order to protect the EuS layer from contamination and to have a normal metal layer providing clean metallic surface necessary to perform STM and STS, Ag is an ideal choice for the top layer, as the proximity effect of diffusive Al/Ag bilayers is well understood and is reliably described by the Usadel equation^47^. The surface RMS roughness of the entire Ag-capped multilayer film is <0.7 nm. The Al(25 nm)/EuS(5 nm)/Ag(5 nm) (Fig. 1a) multilayers have a critical temperature of *T*_c_ ≈ 1.7 K (Supplementary Fig. 7), similar to the critical temperature of the Al(25 nm)/Ag(5 nm) reference sample.

High-resolution transmission electron microscopy (TEM) images (Supplementary Fig. 3) show all films (including the oxide layer between the Al and EuS films) to be nanocrystalline with visible lattice planes within most grains. SQUID magnetometry (Supplementary Fig. 5) and further subgap features (Supplementary Note 7 and Supplementary Fig. 13) are shown in Supplementary Information.

### Scanning tunnelling microscopy

Scanning tunnelling spectroscopy with an IrPt tip is performed in a ^3^He cryostat at 290 mK, which results in differential conductance d*I*/d*V* vs. voltage *V* spectra of the types shown throughout this work. All spectra are normalised to the conductance value far outside the gap. Spectra were recorded in lock-in technique across a 10 MΩ tunnelling gap, the STM set point was set to a tunnelling current of 400 pA at 4 mV tip voltage and the feedback loop was then stopped before voltage sweeps. The STM used to conduct this study was home built in Konstanz^48^ and optimised for high energy resolution spectroscopy at low temperatures^49^. The STM controller used was a commercially available SPM1000 by RHK with a proprietary current amplifier (IVP-300) for STM studies. The amplitude of the AC voltage modulation added to the tip bias voltage was set between 7 and 20 μV at a frequency of 733 Hz.

## Electronic supplementary material

Supplementary Information

## Data Availability

The datasets generated during and analysed during the current study are available from the corresponding authors on reasonable request. The compiled custom computer code applied during the current study is available from the corresponding author W.B. on reasonable request.
