# Supplementary material for: Creation of equal-spin triplet superconductivity at the Al/EuS interface
Source: Nat Commun. 2018 Dec 7;9:5248. doi: 10.1038/s41467-018-07597-w (PMC6286363; doi:10.1038/s41467-018-07597-w)
Supplement: Supplementary file 1 — Supplementary Information [file 41467_2018_7597_MOESM1_ESM.pdf]

SUPPLEMENTARY INFORMATION: CREATION OF EQUAL-SPIN TRIPLET SUPERCONDUCTIVITY AT THE AL/EUS INTERFACE

DIESCH ET AL.

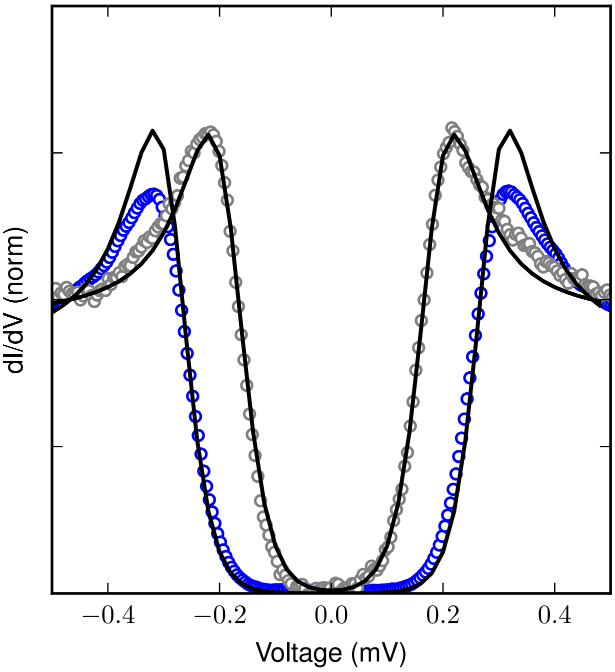

Supplementary Figure 1. **Reference** dI/dV **measurements at** 290 mK **on a** 25 nm **(blue) and a** 240 nm **(grey) Al film**. Both films were capped with 5 nm Ag. Fitting dI/dV spectra calculated based on the Usadel equation (black) results in Δ = 285 µeV and Δ = 185 µeV, respectively, and an effective electron temperature of 295 mK. Data was recorded on sample EuS-3.

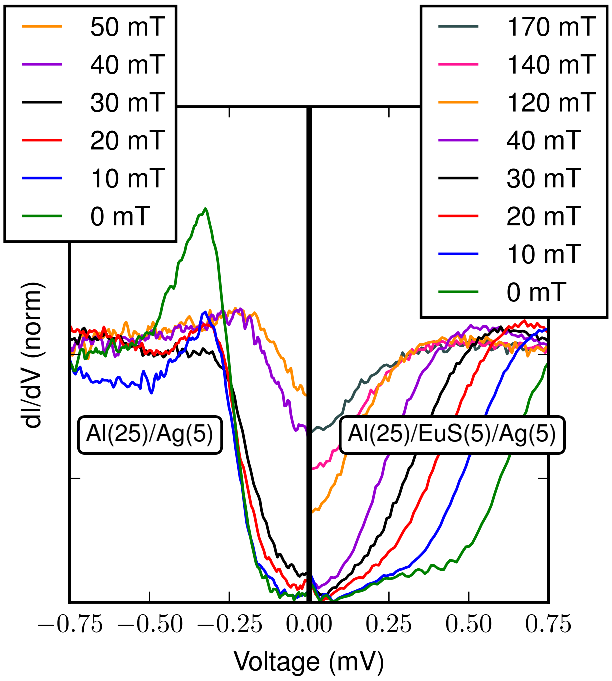

Supplementary Figure 2. **Comparison between Al/EuS/Ag and Al/Ag reference sample.** Magnetic field dependent spectra of a Al(25)/EuS(5)/Ag(5) sample show a higher critical field than the Al(25)/Ag(5) reference sample. Data was recorded on sample EuS-2.

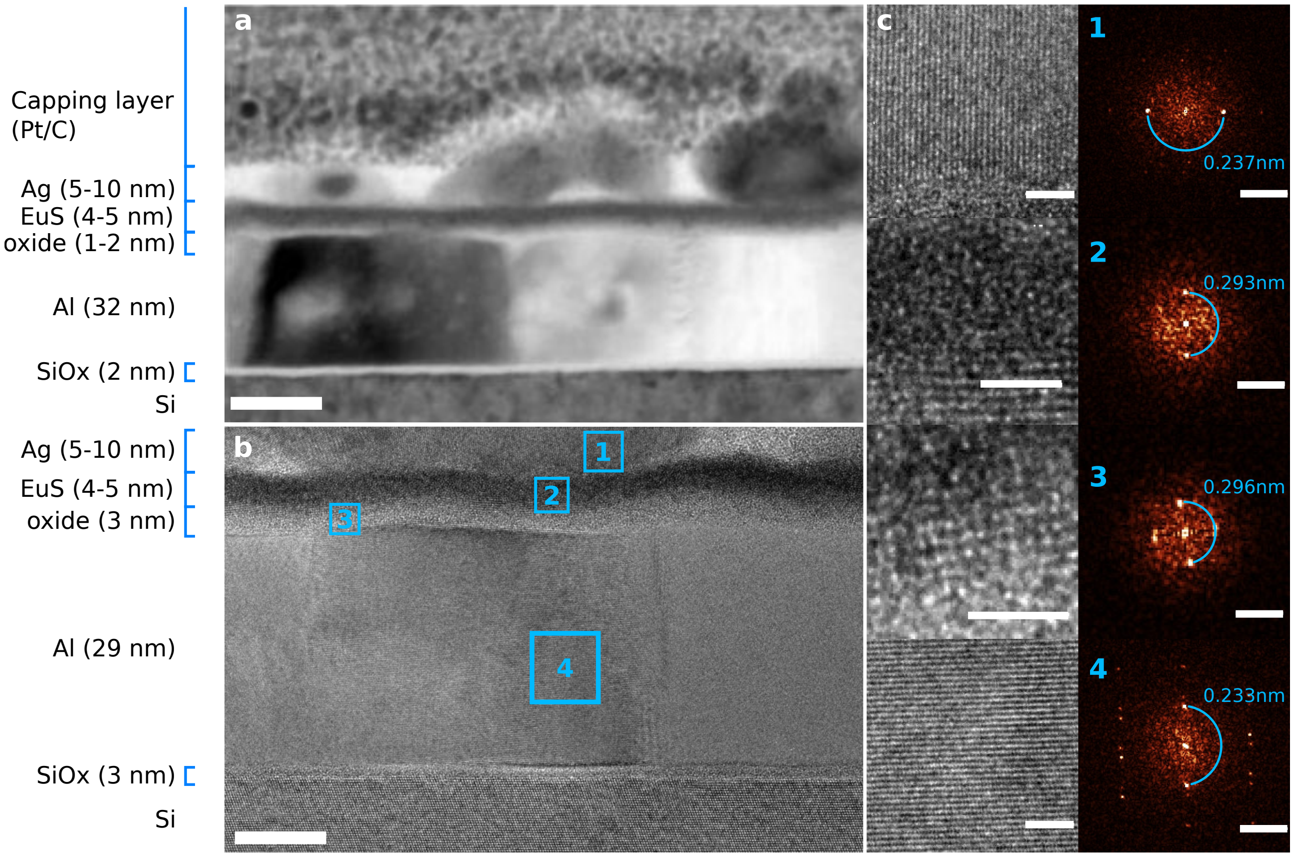

Supplementary Figure 3. **Transmission electron microscopy (TEM) images of a Al(25nm)/EuS(5nm)/Ag(5nm) lamella.** **a,** The scanning transmission electron micrograph shows that growth of the Al and EuS layers are uniform across the sample, with slightly thicker Ag and Al films expected. A bright oxide layer is visible between the Al and EuS films (the scalebar has a length of 10 nm). **b,** The high resolution micrograph shows all films (including the oxide layer) to be nanocrystalline with visible lattice planes for most grains. The oxide layer varies in thickness and shows a gradual transition to the EuS layer and a sharp border to the Al film (the scalebar has a length of 10 nm). **c**, The reciprocal space information (Fast Fourier Transform) shows lattice planes for grains of all films with the expected reflections from the (111) planes for Ag and Al and reflections from the (200) planes for EuS. The reflections in the oxide layer match various Eu oxides, and none of the common reflections of Al oxides (the scalebars have a length of 2 nm for the zoomed topographies and 5 nm^{-1} for the reciprocal space information images). Images were recorded on sample EuS-4.

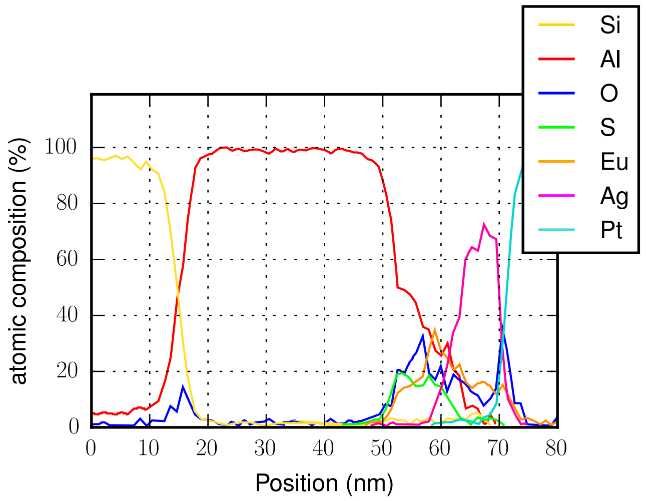

SUPPLEMENTARY FIGURE 4. **Elemental analysis of one Al/EuS/Ag sample by energy dispersive X-ray (EDX) spectroscopy.** The measurement was performed 2.5 months after the sample fabrication. The precence of O between the Al and EuS films is visible. Data was recorded on sample EuS-2.

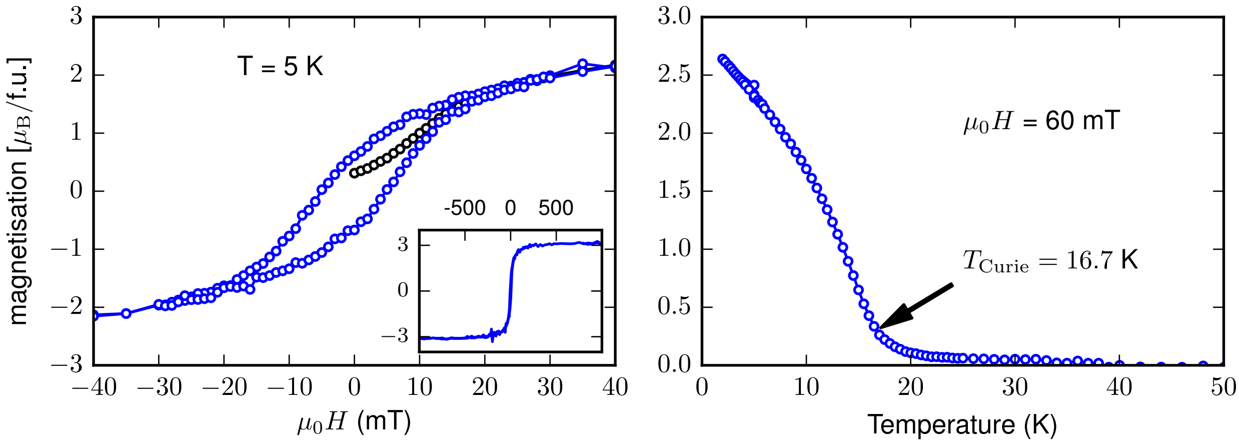

SUPPLEMENTARY FIGURE 5. **SQUID magnetometry measurement in parallel field on one of the Al/EuS/Ag samples (no STS measurements performed on this sample).** The sample is magnetically soft with a coercive field of $\mu_0 H_c \approx 5\,\mathrm{mT}$ and a Curie temperature of $T_{\mathrm{Curie}} \approx 16.7\,\mathrm{K}$, close to the Curie temperature of the bulk material. When zero-field cooled, the film shows almost no spontaneous magnetisation (black curve). The diamagnetic moment of the Si substrate was subtracted from the magnetisation curve. Data was recorded on sample EuS-1.

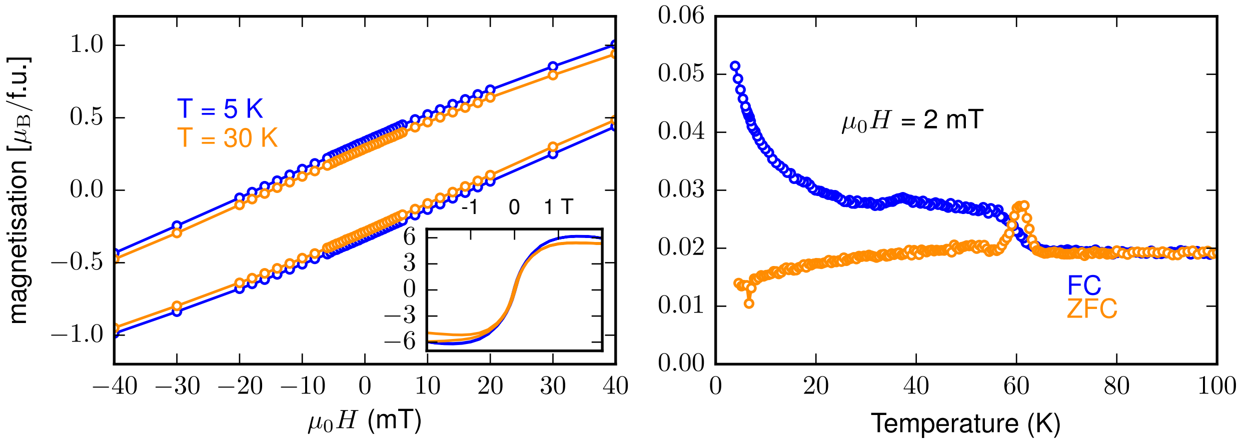

SUPPLEMENTARY FIGURE 6. **SQUID magnetometry in out-of-plane field on Al/EuS/Ag sample EuS-2.** The sample is magnetically soft both at 5 K and at 30 K with a coercive field of 15 mT and two different Curie temperatures of $T_{\mathrm{Curie}_1} \approx 17\,\mathrm{K}$ and $T_{\mathrm{Curie}_2} \approx 63\,\mathrm{K}$, which are close to the exepected Curie temperatures of bulk EuS and EuO, respectively. When zero-field cooled, the film shows a distinct peak in the $M(T)$ curve (orange) around 63 K. Data was recorded on sample EuS-2.

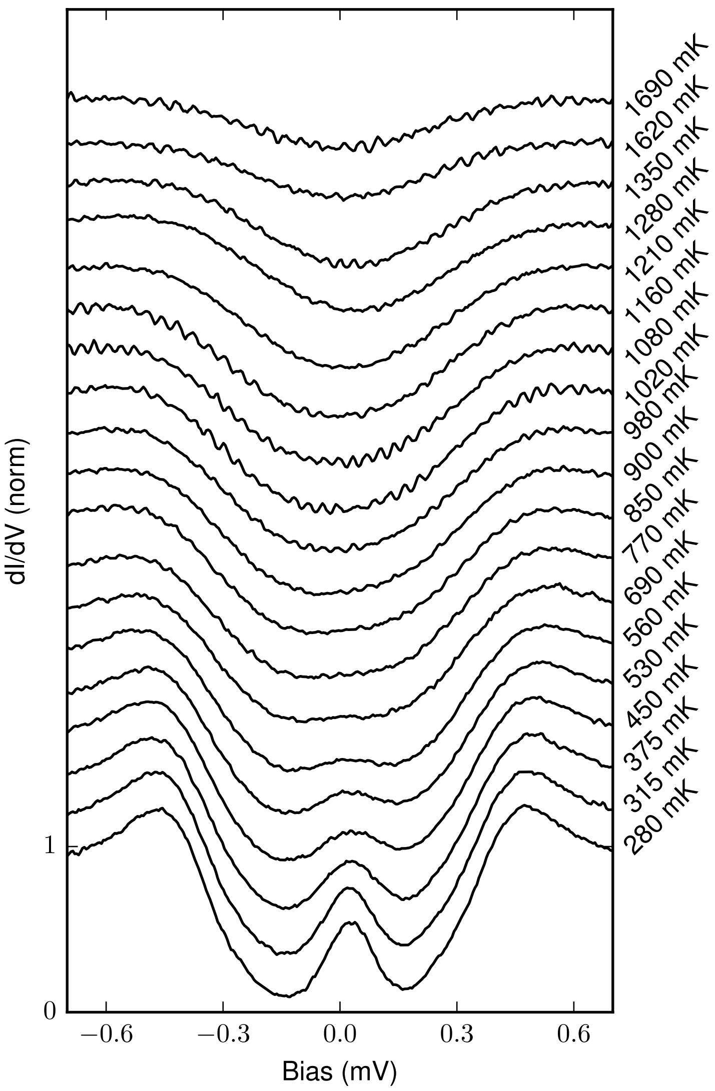

Supplementary Figure 7. **Temperature dependence of the superconducting spectra of an Al(25 nm)/EuS(5 nm)/Ag(5 nm) sample.** The gap includes a zero-bias peak vanishing around 530 mK and shows a second-order phase transition with $T_c \approx 1.7\,K$. All spectra are offset vertically for better visibility. Data was recorded on sample EuS-1.

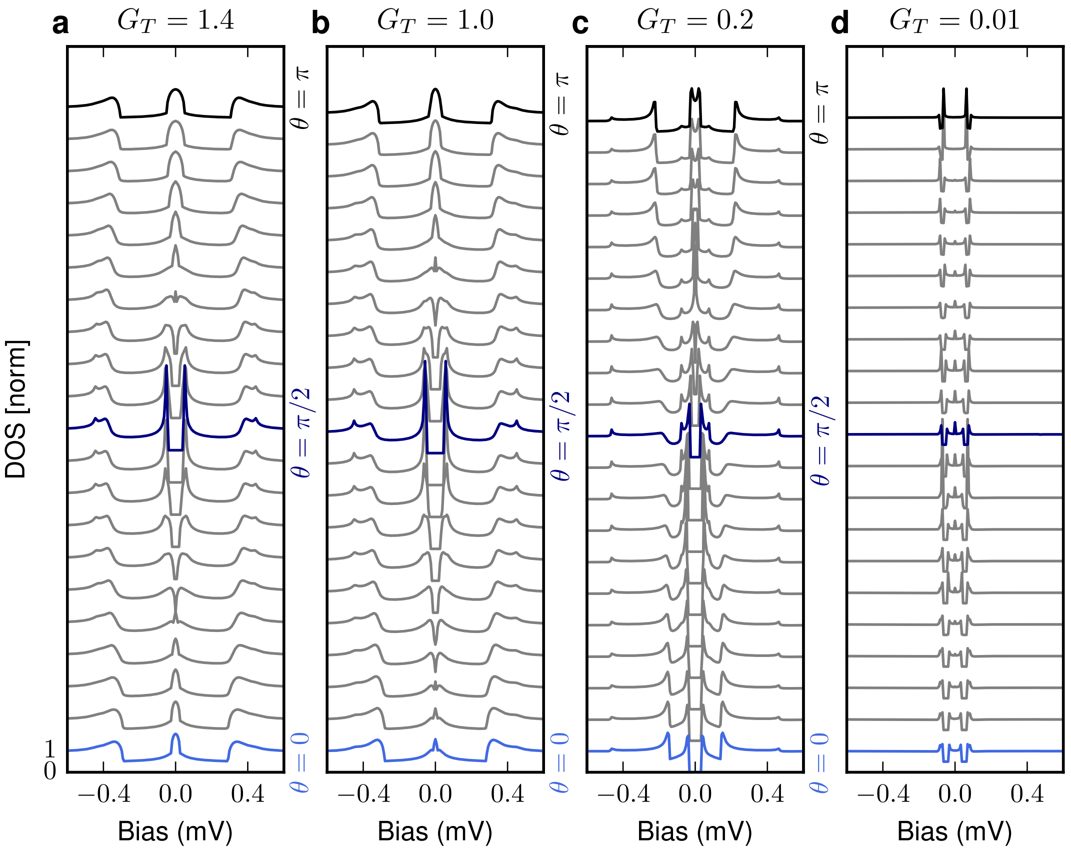

Supplementary Figure 8. **Calculated LDOS at the normal node for different values of the tunnelling conductance G_T.** For low values of G_T, i.e., a well-insulating tunnel barrier, the subgap features get progressively suppressed, while for high values of G_T the subgap features grow in amplitude.

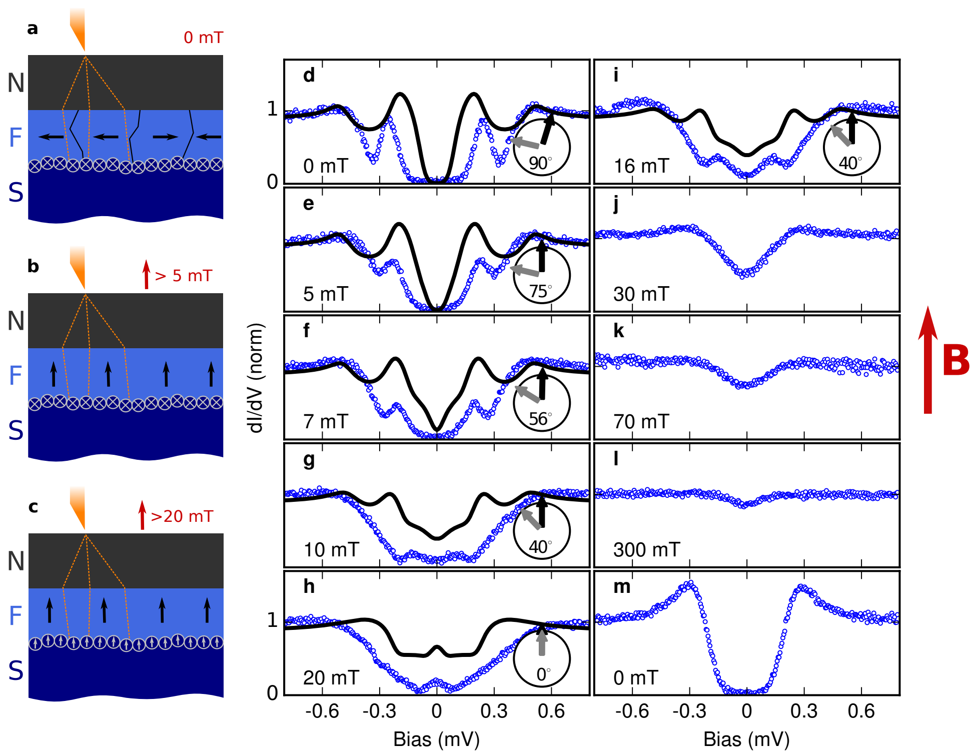

Supplementary Figure 9. **Model of the magnetisation behaviour of the EuS layer for out-of-plane fields.** **a**, The sample in the as-cooled state consists of magnetically soft domains with an overall magnetic moment that is random in direction, and interface moments pinned by impurities which show higher coercive fields. **b**, The internal domains are expected to follow the external magnetic field more readily, aligning at smaller magnetic fields. The interface moments have started to follow the field, but are not aligned yet. **c**, The interface moments only follow the external magnetisation for higher fields. **d - h**, Experimental dI/dV spectra (blue) recorded for the same tunnel contact in varying magnetic fields at 290 mK. A magnetic field sweep is performed to show that the observed curves depend on the magnetisation behaviour of the F layer. The black lines are the calculated differential conductances according to our model. The arrows indicate the fitted relative angle between the different magnetisations. Data was recorded on sample EuS-2.

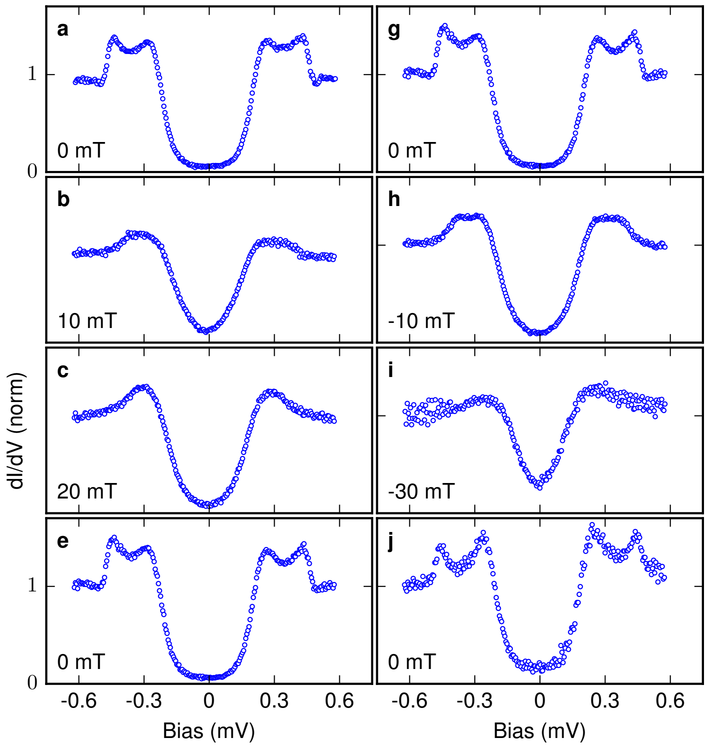

Supplementary Figure 10. **Field dependence of a wide double-peak spectrum, exposed to an out-of-plane magnetic field in both directions.** No hysteresis is detected upon field reversal, and the double peak spectrum returns once the field is turned off. Spectra were recorded at 290mK on an Al(25nm)/EuS(5nm)/Ag(5nm) trilayer. Data was recorded on sample EuS-2.

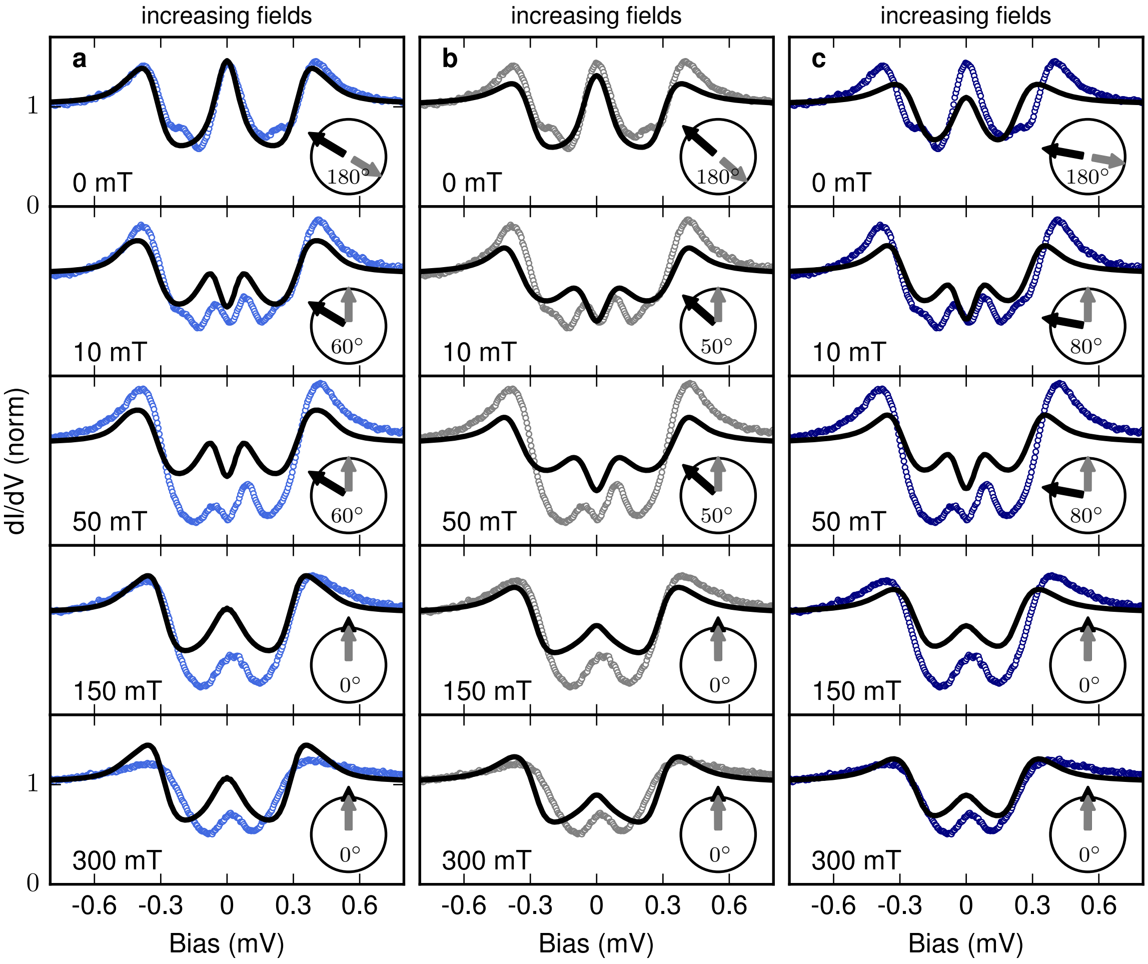

Supplementary Figure 11. **Different parameter combinations for fitting the experimental spectra of Fig. 4. a,** shows the theoretical curves displayed in the main article, **b-c** are show alternative parameter combinations. The numerical parameters for the theoretical curves can be found in Supplementary Tab. 2, experimental spectra are identical to the ones shown in the main article (Fig. 4. For the parameters used, see Supplementary Tab. 2, for LDOS and triplet pairing amplitudes see Supplementary Fig. 12.

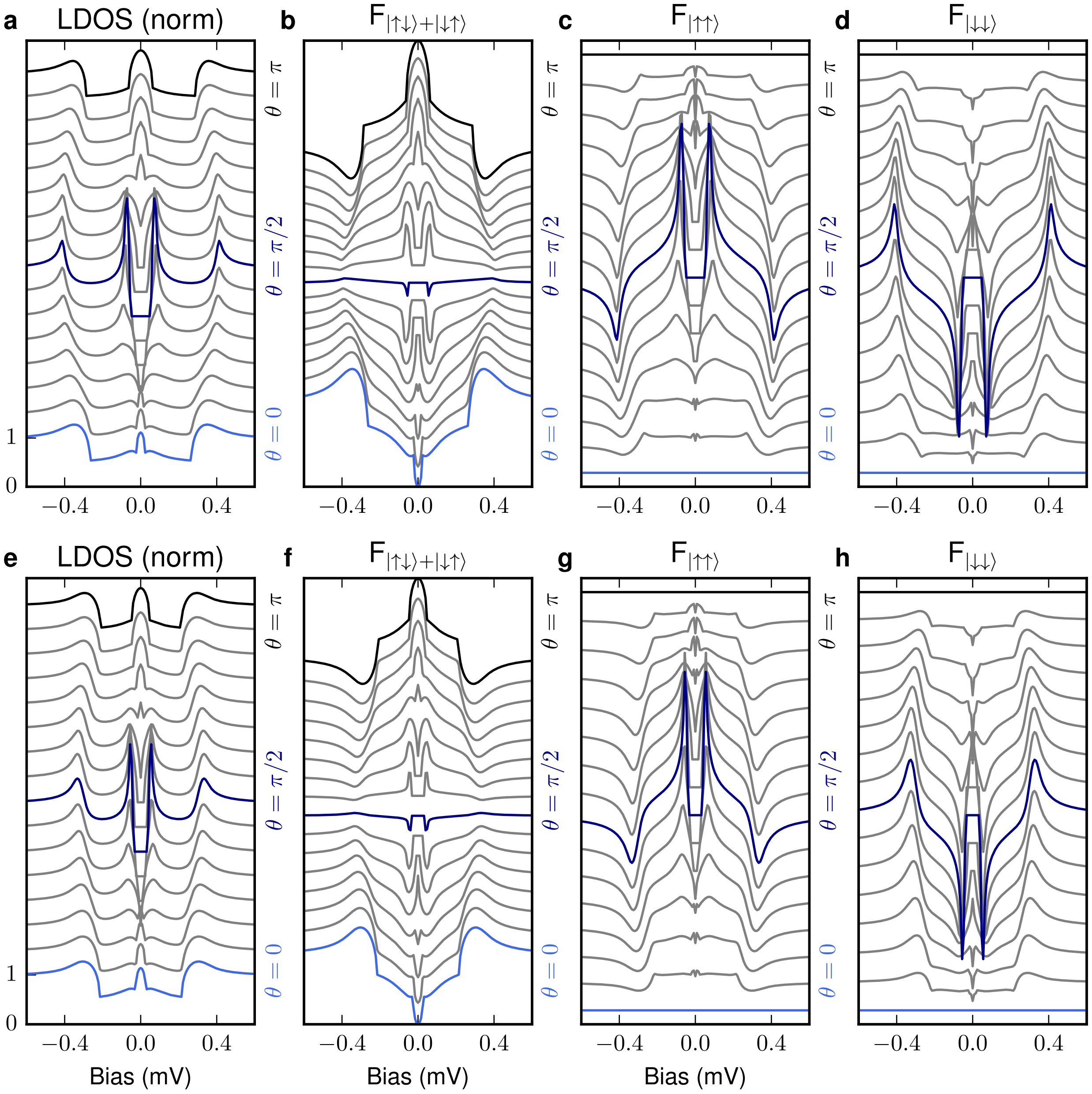

Supplementary Figure 12. **Dependence of the superconducting properties of the S/FI/N system on the magnetic configuration. a-d,** LDOS and triplet pairing amplitudes for the dI/dV spectra shown in 11b, **e-h** the same for the spectra shown in 11c

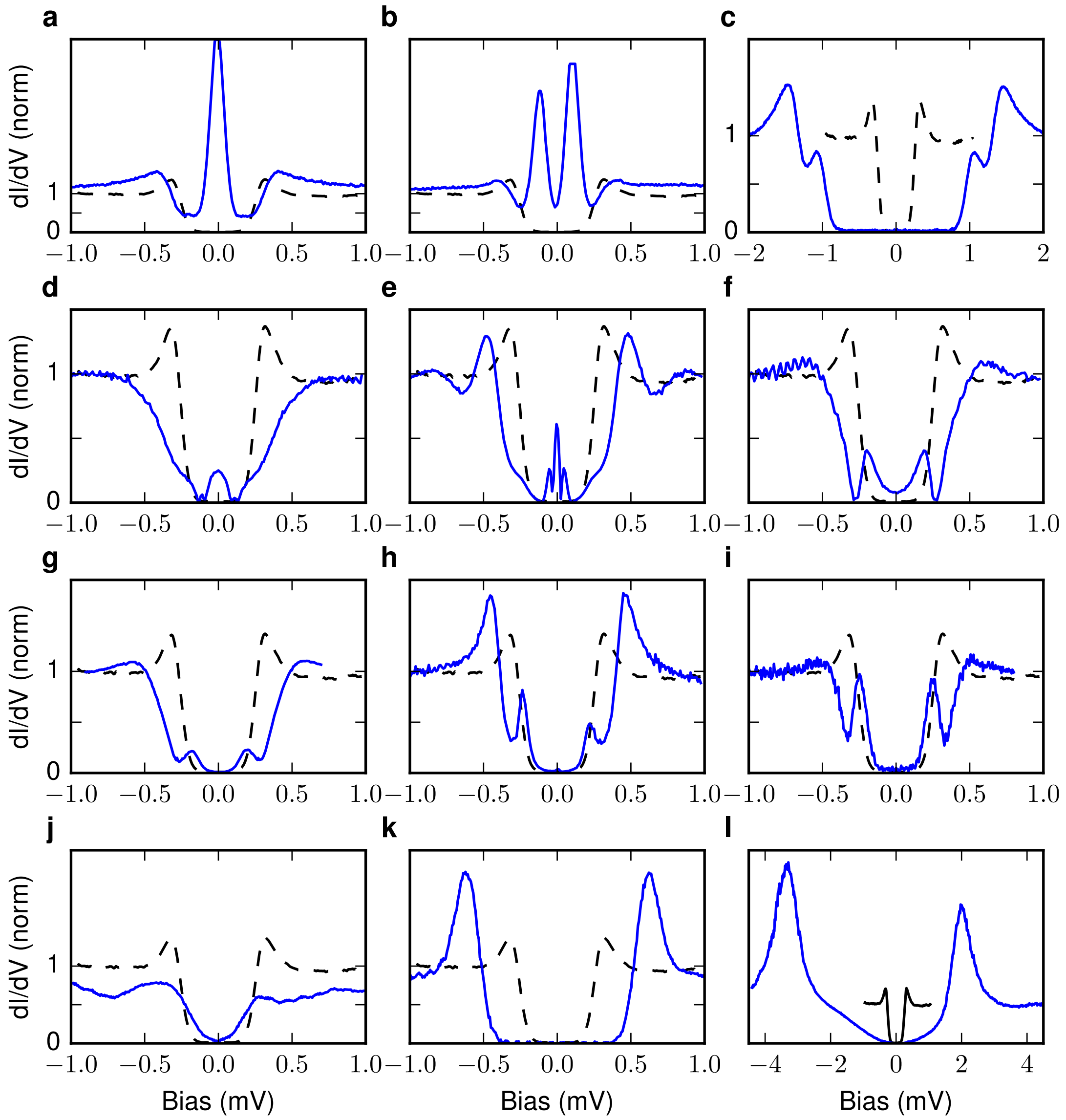

Supplementary Figure 13. **Examples of spectra observed on different Al/EuS/Ag samples (blue) vs. the Al/Ag reference sample (black).** While **a, b** can be described with the circuit theory model given in the main article, **c** features an enlarged energy gap that cannot be explained with it. **d-i** have been measured on a second, nominally identical sample. **d-g** cannot be explained with our current theoretical calculations, but moving to a similar model for very high spin-mixing might allow us to explain these peaks inside the BCS gap in the future **h, i** show a triplet gap similar to the one presented in the main article. **j-l**, spectra found on a third sample, displaying a variety of different widths of the superconducting energy gap. Again, as in **c**, such an increase in the superconducting gap width cannot be explained by our theoretical model.

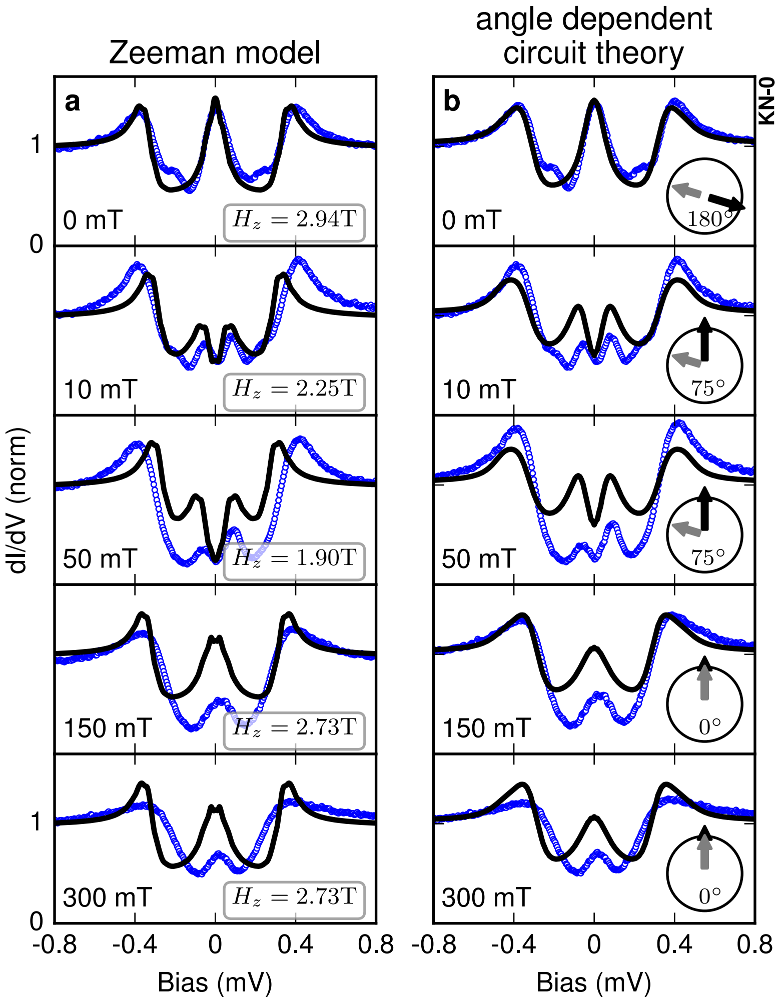

Supplementary Figure 14. **Comparison of Zeeman-spin-split model and circuit theory model.** Selection of experimental dI/dV spectra (blue circles) from Fig. 4 of the main text. **a**, Theoretical modelling with Zeeman-spin-plit LDOS. **b**, Same fits as shown in Fig. 4 of the main text performed with the circuit theory model. While the spectra can also be described qualitatively by the Zeeman model, the resulting fit parameters show a non-monotonous dependence on the field strength. Data was recorded on sample EuS-1.

#	EuS-1	EuS-2	EuS-3	EuS-4	EuS-5
p_0 (mbar)	-	6×10^{-8}	3×10^{-9}	2×10^{-8}	5×10^{-9}
substrate<br>treatment	-<br>-	-<br>-	120 °C,<br>30 min	300 °C,<br>30 min	300 °C,<br>30 min
**layer 1**	Al	Al	Al	Al	Al
thickness d_1 (nm)	25	25	25	25	25
duration t_1 (s)	-	200	720	720	300
rate r_1 (Å s^{-1})	-	1.3	0.35	0.35	
T_S (K)	LN2	LN2	110	77-85	110
p_1 (mbar)	-	3×10^{-6}	8×10^{-7}	-	6×10^{-8}
**layer 2**	EuS	EuS	EuS	EuS	EuS
thickness d_2 (nm)	5	0,2,5,10	0,5,7,10	0,5,7,10	0,2,3.5,5
duration t_2 (s)	-	760	1290	1050	840
rate r_2 (Å s^{-1})	-	0.07	0.08	0.1	0.1
T_S (K)	no LN2	no LN2	400	400	300
p_2 (mbar)	-	6×10^{-6}	6×10^{-6}	5×10^{-6}	2×10^{-6}
**layer 3**	Ag	Ag	Ag	Ag	Ag
thickness d_3 (nm)	5	5	5	5	5
duration t_3 (s)	-	350	420	-	330
rate r_3 (Å s^{-1})	-	0.14	0.12	-	0.2-0.3
T_S (K)	no LN2	no LN2	300	85	300
p_3 (mbar)	-	7×10^{-7}	5×10^{-7}	5×10^{-8}	3×10^{-7}
**TEM**	-	after<br>9 months	-	after<br>2.5 months	after<br>12 days

SUPPLEMENTARY TABLE 1. **Summary of the fabrication parameters of the EuS samples.** Note the addition of an electric heater to the sample holder between the fabrication of EuS-2 and EuS-3, which allows the deposition of the EuS film at 400 K

	units	11a	11b	11c
			12a-d	12e-h
$G_{\mathrm{S}}/(G_{\mathrm{T}}\epsilon_{\mathrm{Th,S}})$	$(k_{\mathrm{B}}T_{\mathrm{c}})^{-1}$	4.1	1.25	0.5
$G_{\mathrm{S}}^{\phi}/G_{\mathrm{T}}$	1	5	1.3	0.45
$G_{\mathrm{N}}/(G_{\mathrm{T}}\epsilon_{\mathrm{Th,N}})$	$(k_{\mathrm{B}}T_{\mathrm{c}})^{-1}$	0.07	0.01	0.035
G^{ϕ}/G_{T}	1	-0.061	-0.05	- 0.01
P_n	1	0.6	0.6	0.4
T_{c}	K	1.84	1.84	1.84
T_{exp}	mK	310	275	330

Supplementary Table 2. **Parameters for the theoretical curves found in Supplementary Fig. 11 and Supplementary Fig. 12.**

1. Supplementary Note: Spectroscopic features of S/F/N multilayers

Two examples for the spectra produced by scanning tunneling spectroscopy (STS) are shown in Supplementary Fig. 1. The measurements were recorded on two different Al films capped with 5 nm Ag, one Al film 244 nm (gray) and the other 25 nm (blue) thick. The recorded $\mathrm{d}I/\mathrm{d}V$ spectra clearly follow the classic shape of the superconducting density of states (DOS), including the BCS gap with its delimiting coherence peaks. The thin film spectrum shown here will serve as a reference sample for all our measurements on Al based superconducting multilayers. Note, that the theory curves shown here are not based on the circuit theory model displayed in later chapters, but on the Usadel equation alone.

Adding a layer of EuS between the Al and Ag results in several significant changes to the $\mathrm{d}I/\mathrm{d}V$ spectra recorded by STS measurements. The first difference between spectra with and without a ferromagnetic layer is the appearance of free states inside the gaps, the "zero-bias peak" and "triplet gap" features described in the main article. The second difference is an increase of the apparent gap width in some cases by up to several hundred µeV (see Supplementary Fig. 2). Comparing with literature [1] on single Al layers (not sandwiched with other layers), the apparent gap size in the Al/Ag layer is smaller because of the strong (negative) proximity effect. In the Al/EuS/Ag system the proximity effect is not so strong, because of the ferromagnetic insulator EuS decoupling the superconductor from the normal metal. Also, the effective thickness of the superconducting Al layer, i.e. the fraction of the layer that becomes superconducting, is reduced compared to the geometrical thickness due to the presence of the ferromagnetic EuS. Both effects would give rise to an enhanced gap size in the Al/EuS/Ag system. Third, the presence of the ferromagnet reliably increased the perpendicular critical field of the multilayer by a factor of at least two. While thin film samples of Al and Ag show a second-order phase transitions to the normal state around out-of-plane fields of 40 mT, a sample including the EuS layer shows the second-order phase transition only at around 170 mT (see Supplementary Fig. 2). When in-plane magnetic fields where applied, the maximum set field of the magnet (500 mT) was not sufficient to suppress superconductivity, as expected for Al films of this thickness ($\mu_0 H_\mathrm{c} \approx 800\,\mathrm{mT}$ is reported for 25 nm films [2]). The fourth difference is that the previously listed phenomena are strongly position dependent for all samples with the EuS interface, while the reference samples consistently show BCS-like spectra everywhere on the sample.

2. Supplementary Note: Sample Characterisation

A series of transmission electron microscopy (TEM) measurements on samples from several different production batches where performed to answer these questions. These microscopy investigations were performed between several months (samples EuS-2 and EuS-4) and only days (sample EuS-5) after sample fabrication and

STS analysis (yet always immediately after the lamella was cut), and the samples were stored in air between the measurements.

Scanning transmission electron microscopy (STEM) images (Supplementary Fig. 3a) show continuous film thicknesses for EuS (dark) and Al, and two oxide layers (bright) above and below the Al film. The contrast within the Al film is a consequence of different crystallites being imaged in different directions with respect to their lattice planes. EDX measurements (see Supplementary Fig. 4) show a clean Al film, but superposition of several components especially at the interfaces between the Al and the EuS layer. Some of these components (Pt and C, the latter being not shown in the EDX analysis) have only been deposited shortly before the focused ion beam (FIB) cutting of the lamella, but we cannot exclude that the EuS film experienced oxidation and some unintended doping during the fabrication process or later, as mentioned above.

High-resolution transmission electron microscopy (HRTEM) images (see Supplementary Fig. 3b) show a certain roughness of this oxide layer resulting in a wavy shape of the EuS and the Ag film on top. Also, lattice planes are visible for all layers when fast Fourier transformation (FFTs) analysis is performed on single grains of the film confirming the polycrystalline growth. While the number of visible reflections in the FFT is low, the peaks of highest intensity (for Al and Ag the reflections of the (111) planes, for EuS the reflections from the (200) planes) can be clearly identified in Supplementary Fig. 3c. The oxide layer shows four prominent reflections indicating a lattice constant of 0.296 nm, which fits closely to the high intensity reflections from the (111) plane of cubic EuO or the (402) plane of monoclinic Eu_2O_3. Because the lattice constants for these compounds are very similar, a closer identification is difficult, but the oxides of Al can be excluded from the list of possible materials. If it was oxidation of the Al, we would expect a fading of the contrast into the Al layer, what we do not observe. Contrarily we observe a sharp contrast change between the Al and the unknown layer and a fading between the unknown layer and the EuS layer. This observation gives an additional indication that the unknown layer is an oxid-containing modification of the EuS layer. While we can cannot easily distinguish EuO and Eu_2O_3 in our TEM analysis, the ferromagnetic nature of EuO should clearly separate it from the non-magnetic properties of Eu_2O_3, which we will show by SQUID magnetometry, revealing a transition around 63 K in addition to the one from the EuS, observed around 17 K. The bulk Curie temperature of EuO is ≈ 69 K, which is expected to decrease in thin films and small grains.

As explained in the main article, we assume that a magnetically harder (relative to the bulk of EuS) interface magnetisation is responsible for the spectroscopic anomalies we observe in our trilayer films. The possible occurrence of ferromagnetic EuO at the interface could provide a possible source for such a magnetic configuration.

The ferromagnetic layer was characterised by SQUID measurements on a 5-nm-thick EuS film, which show that the ferromagnetic transition appears (see Supplementary Fig. 5b), close to the bulk Curie temperature $T_{\mathrm{Curie}} = 16.7\,\mathrm{K}$. The coercive field of the entire magnetic layer is $\mu_0 H_{\mathrm{c}} \approx 5\,\mathrm{mT}$ and the material is magnetically soft, leading to a shallow magnetisation loop (Fig 5a, blue). The saturation magnetisation was around $3\mu_{\mathrm{B}}$ per formula unit, i.e., smaller than the expected value of $7\mu_{\mathrm{B}}$ for a completely saturated film. The fact that the magnetisation curve (black) from zero-field cooled (ZFC) state starts close to $M(H = 0) = 0$ hints at a very uniform domain structure with neighbouring magnetic moments that often cancel each other out, i.e., a distribution with no predominant direction of magnetisation. Hence, a sample starts from a demagnetised state and the domains can be rotated easily and quickly with a relatively low magnetic field. Supplementary Fig. 6 shows equivalent measurements performed on sample EuS-2 after the STS measurements. The hysteresis loop recorded at 30 K shows an only slightly smaller coercive field than the one at 5 K, indicating the existence of a ferromagnetically ordered phase at that temperature. The temperature dependent measurements show in Supplementary Fig. 6b indicate a transition occurring $\approx 63\,\mathrm{K}$, somewhat lower than the Curie temperature of bulk EuO and an increase of the magnetisation below 20 K, consistent with the transition temperature of EuS. These findings support the existence of two magnetically different materials in the sample. From the ratio of the magnetisations we estimate that the material with the higher transition temperature has a much lower volume and might be present in the interface between Al and EuS.

The Al(25 nm)/EuS(5 nm)/Ag(5 nm) multilayer has a critical temperature of $T_{\mathrm{c}} \approx 1.7\,\mathrm{K}$ (see Supplementary Fig. 7), similar to the critical temperature of the Al(25 nm)/Ag(5 nm) reference sample. When recording a temperature dependence on a $\mathrm{d}I/\mathrm{d}V$ spectrum with a zero-bias peak, the anomaly vanishes at around 700 mK.

For more details on fabrication of EuS nanostructures and for more characterisation studies see [3].

3. Supplementary Note: Discussion of the oxide layer

As described in the discussion of our TEM results, an oxide barrier was found between the Al and the EuS layer. If this layer was an insulating, nonmagnetic material, it should significantly reduce the electronic coupling between the Al and Ag layers. In order to investigate the effect of such a low conductance of the tunnelling connector, we performed calculations of the LDOS for different values of the tunnelling conductance G_{T}. Supplementary Fig. 8 shows the results of this study. As visible from those theory curves, lowering of G_{T} first suppresses the features inside the gap, and finally significantly suppresses superconductivity in the Ag layer. This is the expected behaviour for a proximity-coupled system when the coupling strength is decreased. Hence, the existence of a tunnel barrier between the superconductor and the ferromagnetic insulator cannot

explain the appearance of pronounced subgap structures, like the once we observe experimentally. From this result and the fact that we do observe subgap features, we conclude that the oxide layer does not form a strong tunneling barrier, but shares the conductance properties of EuS.

4. Supplementary Note: Magnetic dependence of a triplet gap feature

In the main article, the evolution of a zero bias peak was shown and described with the help of our circuit theory model. Figure 9 displays a magnetic dependence of the differential conductance when the tip is positioned above a region like the one shown in Supplementary Fig. 3d. According to our model, the triplet gap hallmarks the local magnetic moments being oriented noncollinearly (a) with respect to each other. As in the main article, an external magnetic field can be used to first reorient the soft magnetic moments of the bulk (b), and at higher fields, the magnetically harder moments at the S/F interface (c). As displayed in (d)-(m), the spectra follow the same trend as in the main article: the triplet gap evolves into a zero-bias peak as the external field increases, which finally vanishes as the external field begins suppressing the superconductivity of the multilayer.

However, there is a significant difference to the measurement presented in the main article: for this magnetic dependence, an out-of-plane magnetic field was used instead of an in-plane field. Because of that, the sample cannot remain magnetised after the external field is switched off, as the magnetic shape anisotropy does not allow the thin film to remain magnetised in an out-of-plane direction. Thus, as the magnetic field is decreased, the magnetic moments in the thin film layer relax back into an in-plane direction of magnetisation.

The magnetic dependence in Supplementary Fig. 9d-m shows just that behaviour: the spectra are plotted in chronological order, i.e., after the zero-bias peak in (h) was observed at an external field of 20 mT, we decreased the field again to 16 mT and observed the reappearance of the triplet gap in (f). When the field was increased again, suppression of the superconducting state quickly set in at around 30 mT, as expected for out-of-plane fields. No zero-bias peak was encountered, and decreasing the field again now revealed a standard BCS-like gap. This observation again can be explained by the thin film magnetisation not remaining in the out-of-plane direction. Once the external field is removed, all magnetic moments relax into an in-plane direction randomly. This effect results in the formation of many domains, many of which are smaller than they were in the ZFC state. Since these domains don't share an predominant direction of magnetisation, no significant net magnetisation of the probed region remains, and we observe no subgap structure in the tunnel spectrum.

5. Supplementary Note: Symmetry of the magnetic dependence measurements

In the main article, a magnetic field dependence measurement with fields in the positive x-direction is shown (Fig. 4). In the beginning of the experimental work, we routinely swept the field in both polarities, but did not find significant differences and stopped these systematic control measurements after a while to save time. Supplementary Fig. 10 displays an example showing both field directions for another contact. No difference between positive and negative polarity and no hysteresis was observed.

6. Supplementary Note: Numerical parameters

Because the parameter space is large, the theoretical curves presented in the main text are not the only combination of parameters that fit the experimental data well. In Supplementary Fig. 11 we show three sets of curves with different sets of parameters. The calculations based on the circuit theory were carried out with the parameters shown in Supplementary Tab. 2. The parameter set resulting in the spectra in Supplementary Fig. 11a is the data set resulting in the theoretical curves in Fig. 2 and 4 of the main article, Supplementary Fig. 11b,c show completely different parameter sets for comparison. The lower G_S and G_S^ϕ terms result in less pronounced zero-bias peaks, but fit the experimental data better once the triplet gap appears. In order to fit the experimental data at 300 mT, where the external magnetic field results in a significant decrease of the height of the coherence peaks and in a decrease in gap width, several approaches lead to a better fit: introducing increased thermal smearing (by increasing the temperature of the environment), a lower ratio of $G_\mathrm{S}/(G_\mathrm{T}\epsilon_\mathrm{Th,S})$ (shown in Supplementary Fig. 11c) or a stronger proximity effect (higher ratio of $G_\mathrm{N}/(G_\mathrm{T}\epsilon_\mathrm{Th,N})$ all describe the experimental data better for that external magnetic field value.

When changed individually, the parameters in Supplementary Tab. 2 have the following effects on the calculated LDOS (all statements are general trends, changing one parameter always influences many features of the spectra and usually necessitate adjusting other parameters): The polarisation P changes the width of the triplet gap and the zero-bias peak, with lower P resulting in a wider gap and a narrower zero-bias peak. $G_\mathrm{N}/(G_\mathrm{T}\epsilon_\mathrm{Th,N})$ can be understood as a measure for the strength of the proximity effect, where a higher value decreases the gap width and scales down the height of the coherence peaks. $G_\mathrm{S}/(G_\mathrm{T}\epsilon_\mathrm{Th,S})$ and the two spin-mixing terms $G_\mathrm{S}^\phi/G_\mathrm{T}$ and G^ϕ/G_T change the nature of the subgap features entirely. When $G_\mathrm{S}/(G_\mathrm{T}\epsilon_\mathrm{Th,S})$ alone is increased, the width of the triplet gap increases and the amplitude of the zero-bias peak decreases. When $G_\mathrm{S}^\phi/G_\mathrm{T}$ is increased, the zero-bias peak increases in amplitude. When G_S and G_S^ϕ are increased simultaneously, both the features inside the gap and the coherence peaks increase in amplitude. When G^ϕ is decreased, the spectra for $\theta = 0$ and $\theta = \pi$ become more similar and the θ dependence gets increasingly more symmetric.

For all calculations, the superconducting node has a critical temperature $T_c = 1.84\,\mathrm{K}$, which self-consistently results in a superconducting energy gap $\Delta = 1.764 k_B T_c = 280\,\mathrm{\mu eV}$. The experimental temperature T_{exp} was set to between 275 mK and 330 mK in order to achieve the amount of thermal smearing required for a good fit to the experimental data. Both T_c and T_{exp} are realistic for thin film Al (T_c measurement in Supplementary Fig. 7) and the cryostat (an Oxford Instruments Heliox^{TM} VL) used for our experiments.

The pair amplitudes of the resulting triplet states are shown in Supplementary Fig. 12 for the parameter combinations shown in Supplementary Fig. 11b,c. The evolution of the triplet pairing amplitudes behaves in the same way for those two parameter sets as they do for the set used in the main article (Fig. 2), showing that although we cannot make profound predictions about the spin-dependent parameters present in the S/FI interface in our experiments, the message stays the same: there is a direct correlation between the opening of a subgap inside the superconducting energy gap and the presence of equal-spin triplet Cooper pairs.

7. Supplementary Note: other features inside the gap

Supplementary Fig. 13 shows a variety of subgap features measured on different Al(25 nm)/EuS(5 nm)/Ag(5 nm) samples. The spectra depicted in Supplementary Fig. 13a and b are of the kind described in the main article. They fit our theory model well. Supplementary Fig. 13d-i were recorded on a different Al(25 nm)/EuS(5 nm)/Ag(5 nm) sample, and while those spectra also show zero-bias peaks (d,e) and an additional gap-like feature (f,i), these spectra have an important difference to the spectra shown in the main text: the differential conductance reaches $\mathrm{d}I/\mathrm{d}V = 0$ or at least $\mathrm{d}I/\mathrm{d}V \ll 0.5 G_b$ inside the gap at energies higher than the edge of the feature. Although we probed a substantial part of the parameter space, for none of the tested parameter combinations, this behaviour could be obtained from our theoretical model. We currently have no explanation for these features, and will hopefully be able to model them in future work via a similar circuit theory model for very high spin-mixing terms.

For the the type of spectra shown in Supplementary Fig. 13c,h,i, theory curves based on a simple Zeeman field spin-splitting the BCS gap [2] can also result in a good quality of fit. Such a model would not explicitly require the creation of equal-spin triplet pairs. In the case of the spectra displayed in Fig 13h,i, the Zeeman-splitting theory also fails to explain the low $\mathrm{d}I/\mathrm{d}V$ values at energies outside the subgap feature. Summarising, our circuit theory model fits well to all spectra, i.e. those that can and those that cannot be described by a Zeeman splitting of the density of states.

The spectra in Supplementary Fig. 13c,k,l show a large increase in gap width, we observe $\Delta \approx 1.2\,\mathrm{meV}$, $\Delta \approx 0.5\,\mathrm{meV}$ and $\Delta \approx 5.5\,\mathrm{meV}$, respectively. These gap energies are surprising, and we will attempt to explain them in a forthcoming publication.

8. Supplementary Note: Attempt to fit the spectra of Fig. 4 with a Zeeman-model

Supplementary Fig. 14 shows the results of fitting the spectra displayed in Fig. 4 on the main text with a model that assumes a Zeeman-splitted LDOS along the lines of Ref. [2]. To this end, we first fit dI/dV of the Ag/Al sample (see Supplementary Fig. 1). To mimic a spin-split LDOS, we then superpose two of these fitted spectra shifted with respect to each other. Fitting parameters are the relative height of the two parts as well as their splitting. We then translate the calculated splitting into a field strength assuming a g-factor of 2. While qualitatively the shape of the individual spectra can be reproduced with this model, the fitting parameters depend non-systematically on the applied field.

Supplementary References

[1] Court, N. A., Ferguson, A. J. & Clark, R. G. Energy gap measurement of nanostructured aluminium thin films for single Cooper-pair devices. *Superconductor Science and Technology* **21**, 015013 (2008).

[2] Meservey, R. & Tedrow, P. Spin-polarized electron tunneling. *Physics Reports* **238**, 173 – 243 (1994).

[3] Wolf, M., Sürgers, C., Fischer, G., Scherer, T. & Beckmann, D. Fabrication and magnetic characterization of nanometer-sized ellipses of the ferromagnetic insulator eus. *Journal of Magnetism and Magnetic Materials* **368**, 49 – 53 (2014).
